# The Relationship between Power, Sense of Power, and Cognitive Flexibility: An Analysis of Parallel Mediating Effects Based on Reward and Punishment Sensitivity

**DOI:** 10.3390/bs14070513

**Published:** 2024-06-21

**Authors:** Shiyue Cao, Dong Yang

**Affiliations:** Faculty of Psychology, Southwest University, Chongqing 400715, China; shiyuecao@email.swu.edu.cn

**Keywords:** power, sense of power, reward sensitivity, punishment sensitivity, cognitive flexibility

## Abstract

This study utilized a sample of 2052 participants from government and enterprise sectors to explore the distinct effects of power and sense of power on cognitive flexibility. It also delves into how the three dimensions of reward sensitivity and the comprehensive measure of punishment sensitivity mediate this relationship. The key findings are as follows: (1) There is no significant direct correlation between power and sense of power. (2) Both power and sense of power are substantial positive predictors of cognitive flexibility, with middle- and upper-level employees demonstrating significantly greater cognitive flexibility than their lower-level counterparts, and sense of power having a more pronounced positive influence than objective power. (3) Drive and fun-seeking mediate the relationship between sense of power and cognitive flexibility, yet only when sense of power is the independent variable. (4) No mediating effects are observed for the dimensions of reward sensitivity or punishment sensitivity when power is the independent variable. Exploring reward and punishment sensitivity in the context of power’s influence on cognitive flexibility in real organizational settings is of paramount importance. This enhances our understanding of the intricate ways in which power dynamics shape individual behaviors and cognition across diverse cultural landscapes and provides actionable insights for refining organizational management and leadership strategies.

## 1. Introduction

Power is an ancient and significant topic of study in sociology, political science, and various other social sciences. Both scholars and the general public have engaged in enduring discussions about the origins, utilization, and constraints of power [1]. Power plays a crucial role in social interactions, influencing not only an individual’s ability to control resources and affect others, but also forming the cornerstone and core components of leadership [2,3,4,5,6]. Effective leadership cannot exist without this fundamental element [7]. From the perspective of employee personal development, the pursuit and mastery of power are vital drivers for achieving self-improvement and outstanding success within an organization [8]. Therefore, it is evident that the influence of power permeates every aspect of organizational management.

Sense of power is a subjective psychological experience that reflects an individual’s perception of their ability to control resources and influence others. This perception can profoundly affect an individual’s cognition, emotions, and behaviors [2,3,4], and these effects are not necessarily dependent on objective structural power [2,6,9]. Moreover, cultural differences between the East and the West may lead to significant variations in how power is construed by its holders in different cultural contexts [10]. Therefore, exploring the relationship between power and sense of power in a Chinese organizational setting, and understanding how they affect individuals differently, is particularly crucial.

The influence of power and sense of power on individuals may manifest in their behaviors and cognition. On one hand, as a variable within social structures, power can impact cognitive flexibility [11]. From the subjective perspective of psychological perception, sense of power—which involves an individual’s perception of their ability to control resources and influence others—may more accurately predict behaviors and cognitive patterns [2], thereby exerting a profound influence on cognitive flexibility. On the other hand, power, or sense of power, by activating the “behavioral approach system” (BAS), directs individuals to prioritize rewards, positive emotions, automatic cognition, and disinhibited behaviors, generating positive emotions and approach behaviors, and thereby enhancing cognitive flexibility. In contrast, a lack of power or sense of power is associated with punishment, constraints, and threats, activating the “behavioral inhibition system” [12], which could suppress cognitive flexibility. Therefore, it is necessary to explore in real organizational settings the effects of different levels of power and sense of power on individual cognitive flexibility, as well as the mediating roles of reward sensitivity and punishment sensitivity. This research could help optimize organizational management strategies, enhance employee adaptability, and improve leadership effectiveness.

Based on this, the marginal contributions of this paper are mainly reflected in the following aspects: Firstly, this study expands the research on the roles of reward and punishment sensitivity in the influence of power and sense of power on cognitive flexibility within real organizational settings, providing empirical support. Secondly, by applying theories of power and sense of power from Western cultural contexts to the actual cultural environment of China, this helps to more deeply differentiate how power and sense of power influence individual behaviors and cognition across different cultural backgrounds. Finally, by understanding how reward and punishment sensitivity vary across different levels of power and senses of power, organizations can tailor management strategies more precisely, create a more positive work environment, and effectively guide change. This assists in ensuring employees can quickly adapt to new work demands in preparation for a rapidly changing market environment.

## 2. Literature Review and Hypotheses

### 2.1. Definition of Power and Sense of Power

Power is fundamentally a concept of interpersonal relations, characterized as a structural variable where those in power can control others by providing or restricting resources [3]. Galinsky and others [4] view power as a psychological state, suggesting that any activation of power can trigger related concepts and behavioral tendencies. However, power and sense of power are not necessarily directly correlated. Under certain conditions, a sense of power might more effectively predict an individual’s attitudes and behaviors than actual power [2], meaning that even individuals granted explicit power in an organizational system may not necessarily feel it. Moreover, in the context of Chinese culture, which emphasizes collectivism and relational orientations, power may be ascribed different meanings compared to Western contexts, and sense of power may have differing effects on individuals. Currently, the relationship between power and sense of power, as well as their effects in real organizational contexts across different cultural backgrounds, remains unclear.

### 2.2. The Impact of Power and Sense of Power on Cognitive Flexibility

In recent years, social psychologists have delved deeper into understanding how power impacts individual behaviors and cognition from a social cognitive perspective. The situational focus theory of power suggests that individuals with high power, due to their enhanced flexibility and selectivity, can focus more effectively and adapt to environmental changes, thereby improving their cognitive flexibility [11]. On the other hand, individuals with low power tend to pay more attention to unnecessary external information and struggle to identify useful information in complex situations, tending to process all information equally, which affects their cognitive flexibility [13]. Cognitive flexibility, an essential part of executive control, refers to the ability to quickly reconfigure one’s thinking as one switches between different tasks. This ability allows individuals to detach from previous tasks and rapidly construct and adopt new response strategies [14]. Researchers have studied cognitive flexibility across various dimensions, including individual differences [15] and developmental changes [14]. As a social structure variable, power significantly influences cognitive flexibility. From a subjective psychological perspective, a sense of power involves an individual’s belief in their ability to control resources and influence others, which may more accurately predict their behavior and cognitive patterns [2]. Currently, how objective power and subjective sense of power differentially impact cognitive flexibility remains to be further explored.

Based on the above analysis, the following hypotheses are proposed:

**Hypothesis 1:**  *Power and sense of power do not necessarily correlate positively*.

**Hypothesis 2:**  *Power and sense of power have different impacts on cognitive flexibility*.

### 2.3. The Mediating Roles of Reward and Punishment Sensitivity

In the current field of power research, the Approach–Inhibition Theory of Power proposed by Keltner et al. dominates [3,16]. This theory suggests that power activates the behavioral approach system (BAS), guiding individuals to prioritize rewards, positive emotions, automatic cognition, and uninhibited behaviors. In contrast, a lack of power is associated with punishment, constraints, and threats, thereby activating the behavioral inhibition system [12]. Reward sensitivity is linked to the behavioral approach system [12], which, when activated, generates positive emotions and approach behaviors. Reward sensitivity encompasses three core dimensions: reward responsiveness, drive, and fun-seeking. Reward responsiveness refers to the degree of positive reaction to rewarding stimuli; drive to the pursuit of rewarding stimuli; and fun-seeking to the desire for rewarding stimuli [17]. Punishment sensitivity, associated with the behavioral inhibition system, is usually measured by an overall score reflecting the intensity of response to punishment signals or the withdrawal of reward signals, with individuals high in punishment sensitivity displaying more inhibited behaviors [12,18]. Research has shown that individual differences such as the Big Five personality traits, gender, and variations in the brain reward pathway significantly affect different dimensions of reward sensitivity and punishment sensitivity [18,19,20,21]. Particularly, individuals with high reward sensitivity show increased activity in the right frontal lobe and striatum when facing reward cues, thus enhancing performance in tasks related to cognitive flexibility [22,23,24].

Given these insights, it is necessary to explore in real organizational settings how different levels of power and sense of power impact cognitive flexibility, as well as the mediating roles of reward and punishment sensitivity across different dimensions.

Based on this analysis, Hypothesis 3 is proposed: The dimensions of reward sensitivity and punishment sensitivity mediate the relationship between power, sense of power, and cognitive flexibility.

To this end, this study employed a questionnaire method. We targeted employees and mid-to-high-level leaders from the government and enterprises, treating power and sense of power as two separate independent variables. The research aims to explore their differential effects on cognitive flexibility and the mediating roles of reward and punishment sensitivity.

## 3. Materials and Methods

### 3.1. Participants and Procedure

Utilizing cluster sampling methodology, this study targeted employees from the government and enterprises in China. After data verification and the elimination of invalid questionnaires, a total of 2052 valid responses were obtained. The demographic breakdown of respondents was as follows: 1062 males and 990 females; 1147 staff members and 905 middle-to-high-level leaders; and an average age of 39 ± 10.04 years.

### 3.2. Measures

#### 3.2.1. Structural Power

In this study, the categorization of structural power is based on job positions within real organizational contexts. In government departments, officials at the section-head level and above were categorized as middle and senior leaders because of their leadership roles in government; conversely, ordinary civil servants were classified as staff members. In the questionnaires distributed to enterprises, subjects were asked to specify their job positions and select whether they belonged to middle and senior management or were ordinary employees [25].

#### 3.2.2. Generalized Sense of Power Scale

The study employed the General Sense of Power Scale. This scale consists of eight items (e.g., “I can get people to listen to what I say”) and utilizes a 7-point Likert scale. Higher scores indicate stronger levels of perceived power. In this study, the questionnaire’s Cronbach’s α = 0.68.

#### 3.2.3. Behavioral Inhibition System and Behavioral Activation System Scale, BIS/BAS

The behavioral inhibition system and behavioral activation system scale (BIS/BAS) was used to assess reward sensitivity and punishment sensitivity among employees and middle-to-upper-level leaders in government and corporate settings. The scale was revised into a Chinese version by Li Yanzhang, showing good reliability and validity [26]. The Chinese BIS/BAS scale comprises 18 items across two subscales: the behavioral activation system scale (BAS) for measuring reward sensitivity and the behavioral inhibition system scale (BIS) for measuring punishment sensitivity. The BAS includes three subdimensions, which are the reward responsiveness, drive, and fun-seeking scales. Scoring on a 4-point scale, where 1 indicates “strongly agree” and 4 “strongly disagree”, higher scores denote greater sensitivity to reward or punishment. In this study, the Cronbach’s alpha coefficients for the drive, fun-seeking, reward responsiveness, reward sensitivity, and punishment sensitivity subscales were 0.63, 0.67, 0.65, 0.84, and 0.72, respectively. Although the reliability for the drive, fun-seeking, and reward responsiveness scales was relatively low, they were still within an acceptable range.

#### 3.2.4. Chinese Version of Cognitive Flexibility Inventory, CFI

The study utilized the Chinese version of the Cognitive Flexibility Inventory (CFI) [27] to measure individuals’ cognitive flexibility. This questionnaire comprises two dimensions—selectivity and controllability—and it utilizes a Likert 5-point scale for responses, ranging from 1 (“never”) to 5 (“always”). In this research, the Cronbach’s alpha for the questionnaire was 0.86.

### 3.3. Data Analysis

Descriptive statistics, correlational analyses, and reliability and validity tests on the collected data were conducted using SPSS 26.0. The data were further examined through structural equation modeling using Mplus 7.4.

## 4. Results

### 4.1. Common Method Bias Test

To address the potential for common method bias, as the variables of sense of power, behavioral inhibition/activation, and cognitive flexibility were all measured through self-reports, we conducted a Harman’s single-factor test for statistical control [28]. The results indicated that the first factor explained 21.72% of the variance, which is below the critical threshold of 40%. Hence, the common method bias in this study is within acceptable limits.

### 4.2. Descriptive Statistics and Correlation Analysis

The Table 1 presents the mean values and correlation analysis results for power, sense of power, reward sensitivity and its three dimensions (reward responsiveness, drive, and fun-seeking), behavioral inhibition, and cognitive flexibility. The findings indicate significant negative correlations between power and reward responsiveness (*r* = −0.05, *p* < 0.05), drive (*r* = −0.05, *p* < 0.05), fun-seeking (*r* = −0.07, *p* < 0.01), and punishment sensitivity (*r* = −0.07, *p* < 0.01), but a significant positive correlation with cognitive flexibility (*r* = 0.10, *p* < 0.01). Additionally, a sense of power is significantly positively correlated with reward responsiveness (*r* = 0.33, *p* < 0.01), drive (*r* = 0.34, *p* < 0.01), fun-seeking (*r* = 0.39, *p* < 0.01), punishment sensitivity (*r* = 0.42, *p* < 0.01), and cognitive flexibility (*r* = 0.36, *p* < 0.01).

### 4.3. Parallel Mediation Analysis with Sense of Power as the Independent Variable

**Figure 1:**
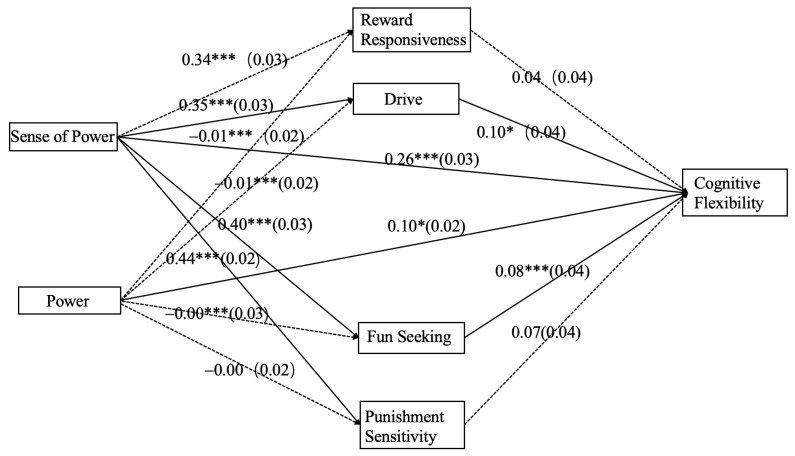
Parallel mediation model diagram. Note: *** *p* < 0.001, * *p* < 0.05. The figures represent standardized coefficients, with standard errors in parentheses. Dashed lines indicate nonsignificant paths. Control variable paths are not displayed for the sake of graphic simplicity.

To investigate the mechanisms by which power and sense of power affect cognitive flexibility, a structural equation model was constructed to further test the mediating effects of reward responsiveness, drive, fun-seeking, and punishment sensitivity. Initially, the total effect of sense of power on cognitive flexibility was tested before examining mediation. The results indicated that after controlling for the effects of gender, age, education level, and positional power, sense of power could significantly and positively predict cognitive flexibility (*β* = 0.364, *SE* = 0.019, *p* < 0.001). With behavioral activation in reward, behavioral activation in drive, behavioral activation in fun, and behavioral inhibition as mediating variables, sense of power as the independent variable, and cognitive flexibility as the dependent variable, and controlling for gender, age, education level, and positional power, a parallel mediation model was constructed. The specific results are shown in Table 2 and Figure 1.

The results from Table 2 and Figure 1 indicate that, after controlling for the effects of gender, age, education level, and positional power, sense of power significantly positively predicts behavioral activation in reward response (*β* = 0.341, *SE* = 0.026, *p* < 0.001), behavioral activation in drive (*β* = 0.348, *SE* = 0.026, *p* < 0.001), behavioral activation in fun-seeking (*β* = 0.404, *SE* = 0.025, *p* < 0.001), and behavioral inhibition (*β* = 0.437, *SE* = 0.023, *p* < 0.001); sense of power also significantly positively predicts cognitive flexibility (*β* = 0.258, *SE* = 0.029, *p* < 0.001). The predictive effect of behavioral activation in reward response on cognitive flexibility was not significant (*β* = 0.035, *SE* = 0.035, *p* = 0.319), whereas behavioral activation in drive significantly positively predicts cognitive flexibility (*β* = 0.095, *SE* = 0.037, *p* = 0.010), as does behavioral activation in fun-seeking (*β* = 0.081, *SE* = 0.037, *p* = 0.030). The predictive effect of behavioral inhibition on cognitive flexibility was not significant (*β* = 0.065, *SE* = 0.039, *p* = 0.099).

Furthermore, the bootstrap method with 5000 resamples was employed to test the parallel mediating roles of behavioral activation in reward response, drive, fun-seeking, and behavioral inhibition between sense of power and cognitive flexibility, controlling for gender, age, education level, and positional power. Detailed results are presented in Table 3 below.

The results from Table 3 indicate that Indirect Effect 1 (sense of power → reward responsiveness → cognitive flexibility) has a value of 0.012 with a bootstrap 95% confidence interval of [−0.012, 0.035], including zero, suggesting that the mediating effect of reward responsiveness between sense of power and cognitive flexibility is not significant. Indirect Effect 2 (sense of power → drive → cognitive flexibility) has a value of 0.033 with a bootstrap 95% confidence interval of [0.008, 0.060], excluding zero, indicating a significant mediating effect of drive between sense of power and cognitive flexibility, accounting for 9.09% of the total effect. Indirect Effect 3 (sense of power → fun-seeking → cognitive flexibility) has a value of 0.033 with a bootstrap 95% confidence interval of [0.003, 0.063], excluding zero, confirming the mediating effect of fun-seeking between sense of power and cognitive flexibility, also accounting for 9.09% of the total effect. Indirect Effect 4 (sense of power → punishment sensitivity → cognitive flexibility) has a value of 0.028 with a bootstrap 95% confidence interval of [−0.006, 0.063], including zero, showing that the mediating effect of punishment sensitivity between sense of power and cognitive flexibility is not significant. The Direct Effect (sense of power → cognitive flexibility) has a value of 0.258, with a bootstrap 95% confidence interval of [0.199, 0.314], excluding zero, indicating a significant direct effect of sense of power on cognitive flexibility, contributing 71.07% to the total effect.

Synthesizing the results from Table 2 and Table 3 and Figure 1, it is evident that drive and fun-seeking mediate the relationship between sense of power and cognitive flexibility, acting as partial mediators. Sense of power can predict cognitive flexibility both by positively forecasting drive and fun-seeking, thereby positively predicting cognitive flexibility, and by directly predicting cognitive flexibility. Moreover, the positive impact of sense of power on cognitive flexibility is significantly greater than the positive impact of power itself (Wald’s χ^2^ = 20.797, *p* < 0.001); the mediating effects of reward responsiveness and punishment sensitivity between sense of power and cognitive flexibility are not supported.

### 4.4. Parallel Mediation Analysis with Power as the Independent Variable

Before examining the mediating effects, the total effect of power on cognitive flexibility was assessed. The results indicated that, after controlling for gender, age, education, and sense of power, power could significantly predict cognitive flexibility positively (*β* = 0.090, *SE* = 0.023, *p* < 0.001), suggesting that middle- and upper-level employees have significantly higher cognitive flexibility compared to ordinary staff.

A parallel mediation model was constructed with reward responsiveness, drive, fun-seeking, and punishment sensitivity as mediating variables, positional power as the independent variable, cognitive flexibility as the dependent variable, and gender, age, education, and sense of power as control variables. Specific results are presented in Table 2 and Figure 1.

The results from Table 2 and Figure 1 indicate that after controlling for gender, age, education, and sense of power, power did not significantly predict reward responsiveness (*β* = −0.012, *SE* = 0.023, *p* = 0.615), suggesting no significant difference in reward responsiveness between ordinary employees and middle- and upper-level management. Power also did not significantly predict drive (*β* = −0.008, *SE* = 0.024, *p* = 0.731), indicating no significant difference in drive between the two groups. Similarly, power’s prediction of fun-seeking (*β* = −0.004, *SE* = 0.023, *p* = 0.867) and punishment sensitivity (*β* = −0.003, *SE* = 0.023, *p* = 0.898) were not significant, showing no significant differences between ordinary employees and higher-level management in these aspects.

However, power could significantly predict cognitive flexibility positively (*β* = 0.092, *SE* = 0.022, *p* < 0.001), indicating that middle- and upper-level management exhibit significantly higher cognitive flexibility compared to ordinary staff. Reward responsiveness did not significantly predict cognitive flexibility (*β* = 0.035, *SE* = 0.035, *p* = 0.319). Drive (*β* = 0.095, *SE* = 0.037, *p* = 0.010) and fun-seeking (*β* = 0.081, *SE* = 0.037, *p* = 0.030) could significantly predict cognitive flexibility positively. In contrast, punishment sensitivity did not significantly predict cognitive flexibility (*β* = 0.065, *SE* = 0.039, *p* = 0.099).

Additionally, the bootstrap method was employed with 5000 resamples to test the parallel mediation of reward responsiveness, drive, fun-seeking, and punishment sensitivity between power and cognitive flexibility, while controlling for the effects of gender, age, education, and sense of power. The specific results are presented in Table 4 below.

The results from Table 4 reveal that Indirect Effect 1 (power → reward responsiveness → cognitive flexibility) has a value of −0.000 with a bootstrap 95% confidence interval ranging from −0.003 to 0.002, which includes zero, indicating that the mediating effect of reward responsiveness between power and cognitive flexibility is not established. Indirect Effect 2 (power → drive → cognitive flexibility) has a value of −0.001 with a bootstrap 95% confidence interval ranging from −0.006 to 0.004, which includes zero, suggesting that the mediating effect of drive is not established either. Indirect Effect 3 (power → fun-seeking → cognitive flexibility) has a value of −0.000 with a bootstrap 95% confidence interval ranging from −0.005 to 0.004, which includes zero, indicating the mediating effect of fun-seeking does not hold. Indirect Effect 4 (power → punishment sensitivity → cognitive flexibility) also has a value of −0.000 with a bootstrap 95% confidence interval ranging from −0.004 to 0.003, which includes zero, showing that the mediating effect of punishment sensitivity is not significant.

Integrating the results from Table 2 and Table 4, and Figure 1, it is clear that the mediating roles of reward responsiveness, drive, fun-seeking, and punishment sensitivity between power and cognitive flexibility are not substantiated. However, power significantly positively predicts cognitive flexibility, with middle- and upper-level employees exhibiting significantly higher cognitive flexibility compared to their ordinary counterparts.

## 5. Discussion

This study explores the impact of power and sense of power on cognitive flexibility within actual governmental and corporate settings, analyzing the mediating roles of reward and punishment sensitivity. It was found that sense of power may have a greater influence on shaping individual behaviors and cognition than structural power. This insight is valuable for precisely designing organizational incentive systems to enhance employees’ cognitive flexibility and, consequently, improve organizational efficacy.

### 5.1. The Relationship between Power and Sense of Power

Contrary to findings by some Western scholars [9,29,30], this study finds that power and sense of power are not necessarily positively correlated; higher positional power does not inevitably accompany a higher sense of power [2,31,32,33]. Consequently, Hypothesis 1 is confirmed. This discrepancy may be due to two reasons: On one hand, existing research on power and sense of power is mostly based on student samples, which may not reflect the complex mechanisms behind power. On the other hand, as a psychological state, sense of power is related to an individual’s perceived ability to control resources and influence others within a specific social structure [24], which does not necessarily rely on objective structural power [2]. In the Chinese cultural context, “power” has two core meanings: one is about measurement and assessment, emphasizing balance and moderation (as MengZi said, “With power, then comes the knowledge of what is heavy and what is light”); the other is the ability to constrain others (as Shenzi said, “To be virtuous but to bend to those who are not worth it is to take power lightly”). In Western languages, “power” refers to the ability to control or influence the thoughts, feelings, or behaviors of others [31]. Such cultural differences may lead to significant variations in how power holders construct the connotations of power and experience and internalize their sense of power across different cultural backgrounds. Therefore, clarifying the distinction and connection between “power” and “sense of power” and exploring the differences between power and sense of power on individual impact are essential for grasping the behavioral patterns within organizations and promoting effective organizational management and leadership strategy development [32].

### 5.2. The Effects of Power and Sense of Power on Cognitive Flexibility

In this research, sense of power and power are posited as independent variables to explore their influence on cognitive flexibility through a structural equation model. Our findings reveal that both sense of power and power significantly predict cognitive flexibility positively, with middle- and upper-level employees exhibiting considerably higher cognitive flexibility compared to their lower-level counterparts, aligning with Guinote [11]. Moreover, the model indicates that the positive impact of sense of power on cognitive flexibility is significantly greater than that of power. Accordingly, Hypothesis 2 is confirmed. The situated focus theory of power suggests that individuals with high power are able to rapidly adjust their thinking and behavioral strategies in response to environmental and goal changes, thereby demonstrating enhanced cognitive flexibility. This attribute is particularly crucial for leaders during organizational transformations. Additionally, possessing power can alter an individual’s cognitive approach, hence boosting their flexibility. In contrast, those with less power are often more affected by environmental changes, focusing excessively on non-essential information, which diminishes cognitive flexibility [11]. At the same time, sense of power as a psychological perception could lead individuals to sense fewer external constraints, enabling them to more freely explore diverse solutions and strategies, thus exhibiting higher cognitive flexibility. In summary, organizations should recognize the differences among individuals in terms of power, sense of power, and cognitive flexibility when selecting talent, placing greater emphasis on assessing and developing employees’ cognitive flexibility. By more effectively considering employees’ reactions and behavioral patterns, organizations can allocate resources more rationally and ensure the maximization of employee potential.

### 5.3. The Mediating Roles of Reward Sensitivity and Punishment Sensitivity

The Approach–Inhibition Theory of Power posits that power activates the behavioral approach system (BAS), triggering reward-associated behaviors and increasing reward sensitivity. Conversely, a lack of power stimulates the behavioral inhibition system (BIS), leading to behaviors that respond to threats and punishment, thus increasing punishment sensitivity [3,16]. Most previous studies have treated power and sense of power as a unified concept to explore their effects on approach and inhibitory behaviors. This study differentiates power and sense of power as independent variables to examine the mediating roles of the three dimensions of reward sensitivity and the overall manifestation of punishment sensitivity in influencing cognitive flexibility.

From the perspective of sense of power, previous research has indicated that individuals with a high sense of power exhibit a stronger activation of the BAS, leading to the effective pursuit of rewards [33]. In our study, only the dimensions of drive and fun-seeking within reward sensitivity acted as mediators between sense of power and cognitive flexibility, while reward responsiveness and punishment sensitivity did not show similar mediating effects. Therefore, Hypothesis 3 is partially confirmed. Specifically, drive refers to the pursuit of rewarding stimuli, and under the influence of sense of power, individuals may feel greater control over resources [24], which not only bolsters their intrinsic motivation but also drives them to pursue rewards and achieve goals. This enhanced drive may further stimulate individuals’ abilities to process new information and adapt to new environments, thereby promoting cognitive flexibility. Fun-seeking relates to the craving for rewarding stimuli and experiences [17]. The positive emotions engendered by a sense of power may amplify the experience and pursuit of rewards, fostering a tendency that encourages diversity and adaptability of thought in the face of new situations and challenges, hence enhancing cognitive flexibility. These findings enrich our understanding of how a sense of power can increase cognitive flexibility by influencing reward processing in individuals. This might also explain why certain individuals are more effective in adapting to and managing new cognitive tasks when their sense of power is heightened, a flexibility that may stem from the activation of internal drives and the pursuit of pleasurable experiences.

Looking at power from a structural perspective, reward responsiveness, drive, fun-seeking, and punishment sensitivity do not exhibit significant mediating effects between power and cognitive flexibility. On one hand, as an objective indicator, positional power may directly affect cognitive flexibility or do so through other unknown variables and psychological processes. Under certain circumstances, sense of power may predict individual attitudes and behavioral responses more effectively than power itself [2], which is evident in self-reported data. On the other hand, individuals with high power may make faster decisions and adapt to new situations due to having greater resources and autonomy, which are not dependent on responses to reward or drives for fun-seeking. The role of punishment sensitivity in affecting cognitive flexibility may be diminished or obscured by factors such as stress, anxiety, or resource scarcity [34,35], and future research could delve into the impact of potential factors like social support, socio-economic status, and cultural differences on the relationship between power and cognitive flexibility.

From above, organizations should recognize the importance of reward sensitivity in enhancing cognitive flexibility among individuals with varying levels of a sense of power. For those employees with a lower sense of power, organizations can boost their intrinsic motivation by enhancing their drive for reward stimuli and their pursuit of pleasure. This not only enables them to more effectively pursue rewards and achieve goals, but also enhances their ability to process new information and adapt to new environments, thereby improving their cognitive flexibility.

### 5.4. The Significance and Limitations of This Study

By exploring the roles of reward and punishment sensitivity in the impact of power and sense of power on cognitive flexibility within real organizational contexts, we can more profoundly understand and distinguish how power and sense of power shape and influence individual behaviors and cognition against the backdrop of different cultures in the East and West. This facilitates organizations in better designing incentive structures to enhance employees’ cognitive flexibility. For instance, for employees or middle and upper management whose positional levels of power do not align with their sense of power, elevating their sense of power by granting them more decision-making authority and a sense of control could be key to unlocking their potential for innovation and flexible problem-solving capabilities. Moreover, understanding the variations in individuals’ reward and punishment sensitivities at different levels of power and senses of power enables organizations to tailor management strategies more precisely, foster a more positive work environment, and effectively lead change to ensure employees can quickly adapt to new work demands in preparation for rapidly changing market conditions.

This study has some limitations that need to be addressed in future research. First, the use of cross-sectional data affects the judgment of the causal relationships between variables. Future studies could adopt longitudinal tracking methods to further explore the causal relationships between power, sense of power, and cognitive flexibility. Second, although this study examined the roles of reward sensitivity and punishment sensitivity in the impact of power and sense of power on cognitive flexibility, the questionnaire format cannot fully and objectively clarify the relationships and mechanisms between these variables. Moreover, self-reported data may be affected by social desirability bias. Hence, future research should employ ingenious experimental methods to obtain more objective and precise data at the psychological and physiological levels [36], while also integrating social, cultural, and organizational contexts to delve deeper into the impact of power and sense of power on cognitive flexibility, as well as the mediating roles of reward and punishment sensitivity.

## 6. Conclusions

This study has reached the following conclusions: (1) There is no significant positive correlation between power and sense of power. (2) Both power and sense of power can significantly positively predict cognitive flexibility, with the cognitive flexibility of middle- and upper-level employees being notably higher than that of ordinary employees, and sense of power having a greater positive effect on cognitive flexibility than power itself. (3) When sense of power is the independent variable, only drive and fun-seeking show mediating effects between sense of power and cognitive flexibility among the three dimensions of reward sensitivity and overall punishment sensitivity. (4) With power as the independent variable, none of the dimensions of reward sensitivity or punishment sensitivity mediate between power and cognitive flexibility.

## Figures and Tables

**Table 1:** Descriptive statistics and correlation analysis for each variable (*N =* 2052).

	1	2	3	4	5	6	7
1 Power	1						
2 Sense of power	−0.06 *	1					
3 Reward responsiveness	−0.05 *	0.33 **	1				
4 Drive	−0.05 *	0.34 **	0.76 **	1			
5 Fun-seeking	−0.07 **	0.39 **	0.68 **	0.72 **	1		
6 Punishment sensitivity	−0.07 **	0.42 **	0.68 **	0.71 **	0.78 **	1	
7 Cognitive flexibility	0.10 **	0.36 **	0.28 **	0.30 **	0.30 **	0.31 **	1
*M*	1.44	31.29	11.65	11.48	13.93	13.54	66.12
*SD*	0.50	5.99	1.72	1.66	2.10	2.23	8.24

Note: ** *p* < 0.01, * *p* < 0.05.

**Table 2:** Results of the parallel mediation model.

Outcome Variables	Predictor Variables	*β*	*SE*	*t*	*p*
Reward responsiveness	Gender	0.132	0.021	6.244	<0.001
	Age	−0.017	0.022	−0.788	0.431
	Education background	0.049	0.022	2.199	0.028
	Power	−0.012	0.023	−0.503	0.615
	Sense of power	0.341	0.026	13.237	<0.001
*R* ^2^		0.129			
Drive	Gender	0.062	0.021	2.897	0.004
	Age	−0.061	0.029	−2.106	0.035
	Education background	0.023	0.024	0.952	0.341
	Power	−0.008	0.024	−0.344	0.731
	Sense of power	0.348	0.026	13.527	<0.001
*R* ^2^		0.126			
Fun-seeking	Gender	0.111	0.021	5.421	<0.001
	Age	−0.096	0.022	−4.436	<0.001
	Education background	0.019	0.022	0.877	0.380
	Power	−0.004	0.023	−0.167	0.867
	Sense of power	0.404	0.025	16.000	<0.001
*R* ^2^		0.178			
Punishment sensitivity	Gender	0.071	0.020	3.530	<0.001
	Age	−0.121	0.024	−5.042	<0.001
	Education background	0.053	0.022	2.363	0.018
	Power	−0.003	0.023	−0.129	0.898
	Sense of power	0.437	0.023	18.675	<0.001
*R* ^2^		0.208			
Cognitive flexibility	Gender	−0.066	0.021	−3.187	0.001
	Age	0.075	0.022	3.427	0.001
	Education background	0.031	0.022	1.394	0.163
	Power	0.092	0.022	4.115	<0.001
	Sense of power	0.258	0.029	8.892	<0.001
	Reward responsiveness	0.035	0.035	0.997	0.319
	Drive	0.095	0.037	2.563	0.010
	Fun-seeking	0.081	0.037	2.164	0.030
	Punishment sensitivity	0.065	0.039	1.651	0.099
*R* ^2^		0.199			

**Table 3:** Bootstrap test results for parallel mediation effects.

	Effect Value	SE	Bootstrap 95% Confidence Interval	
			Lower Limit	Upper Limit
Direct Effect	0.258	0.029	0.199	0.314
Indirect Effect 1	0.012	0.012	−0.012	0.035
Indirect Effect 2	0.033	0.013	0.008	0.060
Indirect Effect 3	0.033	0.015	0.003	0.063
Indirect Effect 4	0.028	0.017	−0.006	0.063

Note: Direct Effect = sense of power → cognitive flexibility; Indirect Effect 1 = sense of power → reward responsiveness → cognitive flexibility; Indirect Effect 2 = sense of power → drive → cognitive flexibility; Indirect Effect 3 = sense of power → fun-seeking → cognitive flexibility; Indirect Effect 4 = sense of power → punishment sensitivity → cognitive flexibility.

**Table 4:** Bootstrap test results for parallel mediation effects.

	Effect Value	SE	Bootstrap 95% Confidence Interval	
			Lower Limit	Upper Limit
Direct Effect	0.092	0.022	0.048	0.134
Indirect Effect 1	−0.000	0.001	−0.003	0.002
Indirect Effect 2	−0.001	0.002	−0.006	0.004
Indirect Effect 3	−0.000	0.002	−0.005	0.004
Indirect Effect 4	−0.000	0.002	−0.004	0.003

Note: Direct Effect refers to power predicting cognitive flexibility; Indirect Effect 1 is power influencing cognitive flexibility through reward responsiveness; Indirect Effect 2 is power influencing cognitive flexibility through drive; Indirect Effect 3 is power influencing cognitive flexibility through fun-seeking; Indirect Effect 4 is power influencing cognitive flexibility through punishment sensitivity.

## Data Availability

The datasets generated during and/or analyzed during the current study are available from the corresponding author on reasonable request.
